# Manual Annotation Studio (MAS): a collaborative platform for manual functional annotation of viral and microbial genomes

**DOI:** 10.1186/s12864-021-08029-8

**Published:** 2021-10-09

**Authors:** Matthew R. Lueder, Regina Z. Cer, Miles Patrick, Logan J. Voegtly, Kyle A. Long, Gregory K. Rice, Kimberly A. Bishop-Lilly

**Affiliations:** 1 Genomics and Bioinformatics Department, Biological Defense Research Directorate, Naval Medical Research Center, Fort Detrick, MD USA; 2 Leidos, Reston, VA USA; 3 Mount St. Mary’s University, Emmitsburg, MD USA

**Keywords:** Genome annotation, Gene annotation, Functional annotation, Manual annotation, Phage annotation, Bioinformatics, High-performance computing, Phage, Microbial genomics

## Abstract

**Background:**

Functional genome annotation is the process of labelling functional genomic regions with descriptive information. Manual curation can produce higher quality genome annotations than fully automated methods. Manual annotation efforts are time-consuming and complex; however, software can help reduce these drawbacks.

**Results:**

We created Manual Annotation Studio (MAS) to improve the efficiency of the process of manual functional annotation prokaryotic and viral genomes. MAS allows users to upload unannotated genomes, provides an interface to edit and upload annotations, tracks annotation history and progress, and saves data to a relational database. MAS provides users with pertinent information through a simple point and click interface to execute and visualize results for multiple homology search tools (blastp, rpsblast, and HHsearch) against multiple databases (Swiss-Prot, nr, CDD, PDB, and an internally generated database). MAS was designed to accept connections over the local area network (LAN) of a lab or organization so multiple users can access it simultaneously. MAS can take advantage of high-performance computing (HPC) clusters by interfacing with SGE or SLURM and data can be exported from MAS in a variety of formats (FASTA, GenBank, GFF, and excel).

**Conclusions:**

MAS streamlines and provides structure to manual functional annotation projects. MAS enhances the ability of users to generate, interpret, and compare results from multiple tools. The structure that MAS provides can improve project organization and reduce annotation errors. MAS is ideal for team-based annotation projects because it facilitates collaboration.

**Supplementary Information:**

The online version contains supplementary material available at 10.1186/s12864-021-08029-8.

## Background

Genome annotation is the process of locating and labelling functional regions within a genome. The location of functional regions is determined in a process called gene calling. Typically, this is performed using automated software tools such as Glimmer [1], GeneMark [2], or Prodigal [3] which use statistical modelling to predict the presence and location of each gene. After gene calling, a descriptive label and metadata are provided for each predicted gene in a process called functional annotation. A fundamental use of annotation is to discover and characterize genes of interest. For example, human genome annotation may be used for discovering disease-causing variants [4], prokaryotic annotation for discovering genes responsible for pathogenicity or antibiotic resistance [5], and viral genome annotation for discovering potential targets for therapeutics [6]. There have been several efforts to completely automate the process of microbial genome annotation; Systems such as RAST (Rapid Annotation using Subsystems Technology) [7], Prokka [8], and the Prokaryotic Genome Annotation Pipeline (PGAP) [9] can predict gene locations and provide a functional annotation with the click of a button. The previously described annotation use cases require detailed and accurate annotations. While these automated tools provide convenience, manual curation is needed for more accurate and detailed annotations [10, 11].

Optimal manual functional annotation involves using an ensemble approach, where multiple tools and databases are used and results from each are compared and contrasted [11]. There are many examples in the literature which use this approach to improve upon previous annotations [12–15]. This approach is typically accomplished by submitting separate searches to multiple disparate web servers and comparing results by clicking between tabs then saving the final annotation in a text file or spreadsheet. With this method, search results are transient, there is significant overhead to start the searches, and direct comparison of results is more difficult.

To improve this process, we developed Manual Annotation Studio (MAS), a software tool that assists users throughout the process of manual functional annotation. MAS is a user-deployable web server that provides an interface for creating and editing annotations and collecting and reviewing evidence for the function of genes. MAS allows users to perform an ensemble of homology searches at the click of a button and provides interactive visualizations of search results and of the genome being annotated. It organizes annotations into a structured database, allowing them to be easily searched. It also facilitates team efforts by allowing multiple users to work on the same data concurrently, and encourages consistency of annotations by automatically generating a searchable BLAST database of previously annotated proteins. MAS can be run offline and can utilize high-performance computing (HPC) clusters through SGE (Oracle Grid Engine) or SLURM (Simple Linux Utility for Resource Management).

## Results and discussion

### Comparison to existing tools

Current commonly used annotation editing tools include GenDB [16], Apollo [17], DNA Master [18], and CLC Genomics Workbench. Here, we define an annotation editing tool as any tool which allows users to import a genomic sequence, to manually label regions within the genomic sequence with functional information, and to export the fully annotated sequence.

GenDB is an annotation editing tool which allows users to view results for multiple functional annotation tools. GenDB supports the annotation of prokaryotic genomes and more recently, the annotation of eukaryotic genomes through GenDBE. Similar to MAS, it performs automated gene calling, supports multiple functional annotation tools, and saves annotations and tool results to a database. In addition, GenDB performs automated gene calling. GenDB was originally created by Bielefeld University as an open source alternative to the commercial and/or closed source annotation tools. However, GenDB is currently maintained by the University of Giessen and it is no longer open source. GenDB is offered as a web service hosted on infrastructure provided by the University of Giessen and accessed by users through a web browser. To get an account to use GenDB, users agree to either an academic or a commercial partnership with the university. The university will then upload genomes to the server on behalf of the user, run the gene calling and functional annotation tools, and backup user data. In contrast, MAS is fully open source, allows users to keep their data completely private, and can be used without intervention from an outside group. However, MAS requires users to install and run it on their own infrastructure.

Apollo is an open-source annotation editing platform. Like MAS, it supports collaboration and can be installed locally with Docker. However, Apollo focuses on gene structure annotation rather than functional annotation. Gene structure annotation is the process of identifying the location of genes, alternative splicing sites (exons and introns), and regulatory sequences. MAS does not focus on this aspect of annotation and instead predicts gene coordinates with automated gene-callers or lets users upload custom coordinates. Functional annotation within Apollo is performed by exporting protein sequences as FASTA files and using tools outside of Apollo to find homology. Because MAS and Apollo focus on different aspects of annotation, these tools can be used in conjunction. In situations where automated gene calling is not accurate enough, Apollo can be used to determine the coordinates of coding sequences, these coordinates can be imported into MAS, and MAS can be used to functionally annotate the genome.

DNA Master is a genome annotation tool that has become a popular option for bacteriophage genome annotation because of its use in the SEA-PHAGES project [19]. It is available as an executable for Windows machines and can be used for structural and functional annotation. DNA master allows users to adjust the coordinates of coding sequences based on the results of multiple gene callers and evidence from sequence homology. DNA Master provides a form for editing functional annotations and is integrated with NCBI’s BLAST API, which provides results from BLAST searches against NCBI’s non-redundant protein (nr) database. However, functional annotations based on a single tool and database are not as accurate or descriptive as functional annotations based on evidence from multiple sources. DNA Master runs locally, on a single machine, and does not provide a framework for collaborative annotation. As with Apollo, CDS coordinates which have been refined using DNA Master’s manual structural annotation capabilities can be imported into MAS.

CLC Genomics workbench supports a wide array of genomic analyses, including manual genome annotation. It supports basic automated gene calling and also allows users to add annotations to manually selected genomic regions. The integration of BLAST and HMMER provides support for functional annotation. However, CLC Genomics Workbench is a commercial software product that must be purchased to use.

### Genomes, features, and annotations

MAS manages three main types of data: *genomes, features*, and *annotations*. This data is created when a user uploads a genomic sequence to MAS. Each *genome* in MAS contains the genomic sequence of an organism and is associated with a number of *features*. A *feature*, on the other hand, is specific to a single *genome* and describes the location of a coding sequence (CDS), tRNA, or repeat region. Further information about a *feature* is contained within its associated *annotation*. An *annotation* contains an accession, a label, a sequence, notes fields, a flag, and an optional user assignment. Accessions are assigned to *annotations* automatically while labels, notes fields, and flags are assigned during the manual annotation process. The sequence of an *annotation* is unique to it (i.e. only one *annotation* exists per unique protein) and can be a protein sequence, an RNA sequence, or a DNA sequence depending on the type of *feature* with which it is associated. Two *features* will be associated with the same *annotation* if they have identical sequences. For example, if two phage genomes are uploaded and they both encode a protein with an identical sequence, then each phage will have their own *feature* but will share an *annotation*.

### Genome upload

Genome sequences can be provided to MAS as a single-sequence FASTA file. Upon upload, a *genome* and associated *features* and *annotations* will be created and saved to the MAS database. Genome sequences can be uploaded through the phage genome upload, bacterial genome upload, or custom upload tabs. If a genome is uploaded through the phage or bacterial genome upload tabs, gene-calling is automated with Glimmer (v3.02) to find CDSs and tRNAscan-se (v2.0.6) to find tRNA genes. When uploading a phage genome, users can choose to re-orient the genome from the terminase gene. Using this option is a best practice when genomic termini cannot be determined based on packaging strategy and ensures similar genomes start from the same position, facilitating comparative genomics [20]. If this option is selected, MAS will automatically search all CDSs using blastp against a database of terminase proteins. If there is enough evidence of a terminase gene, it will automatically re-orient the genome based on that genomic position. Whereas if the genome contains both small and large terminase subunits, the genome will be re-oriented to start from whatever subunit is upstream. If the phage genome contains direct terminal repeats, the user can enter the size of the repeats and *features* will be created to represent them. The custom upload tab allows users to upload their own gene-calls. When uploading a genome sequence, the user has the option to assign all new *annotations* created as a result of the upload to a specific user.

### The result viewer

**Fig. 1:**
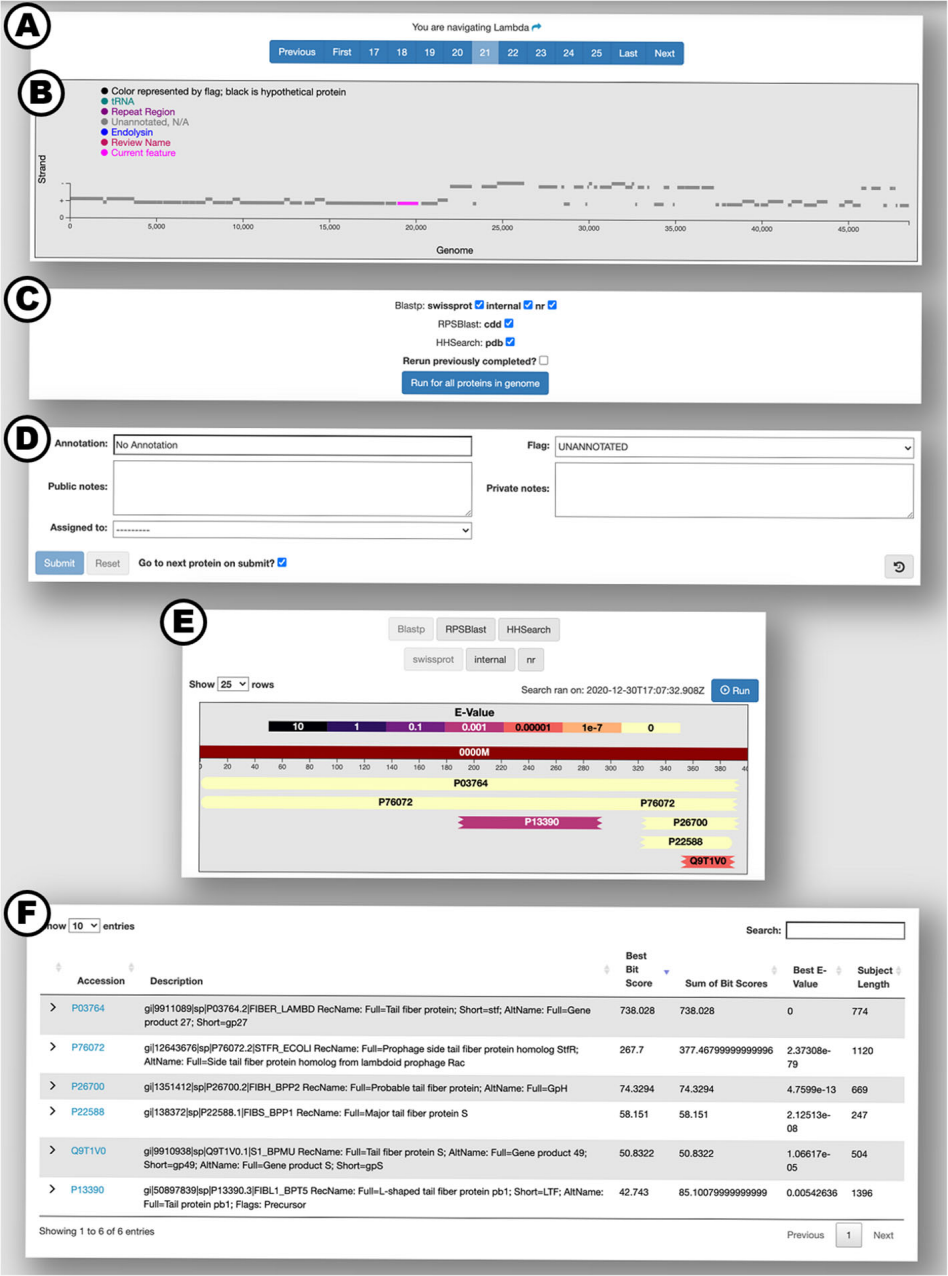
The result viewer page with the ‘navigate by genome’ navigation option selected. **A** The navigation pane gives the user the ability to navigate through the selected group of Annotations. Here, CDS 21 of phage lambda is selected. **B** The genome visualization depicts each feature in the selected genome at its genomic coordinates. The color of the feature is determined by the flag assigned to the annotation associated with the feature (e.g. blue for endolysin, purple for repeat region). Hovering over a feature will display information about the feature’s annotation and clicking it will navigate to that annotation. **C** When the ‘navigate by genome’ option is selected, users have the ability to initiate homology searches for all proteins in the selected genome. **D** The annotation form allows users to edit the attributes of an annotation. The history of an annotation can be displayed by clicking the “show history” button in the bottom right. **E** The result visualization pane allows users to select which tool and database for which they would like to view results. Proteins/domains returned from the selected search are shown as colored blocks aligned against the query sequence. The color of the block represents the statistical significance of the alignment (e.g. black for e-value 10 to light yellow for e-value 0). Clicking on a block highlights the corresponding result in the result table. Single searches are initiated from this pane. **F** The result table contains additional information for each hit returned by the selected homology search. The name and length of the returned subject/target are given, along with additional statistical metrics. Users can see the entire alignment in text format using the drop-down arrows. Links are available to navigate to further information about the subject/target in its native database

The result viewer can be accessed through one of the result navigation options or through links in the genome and annotation lists and details views. This is where homology searches are launched, annotations are edited, and search results are visualized. One annotation is displayed at a time but the navigation pane allows users to switch to different annotations easily (Fig. 1A). The function of the navigation pane depends on the selected navigation option. There are three navigation options: *navigate by flag*, *navigate by assignment*, and *navigate by genome*. If the *navigate by flag* option is being used, the navigation pane will guide the user through annotations assigned to the selected flag option, sorted by accession. If the *navigate by assignment* option is being used, the user will be able to navigate through all annotations assigned to a specific assigned user, also sorted by accession. Finally, if the *navigate by genome* option is selected, the navigation pane can be used to navigate through annotations associated with the selected genome, sorted by their genomic coordinates. If the selected genome has fewer than 1000 features, an interactive visualization of the genome will appear in the navigation pane (Fig. 1B). This visualization can be used to navigate through the annotations assigned to the selected genome.

Homology searches can be launched on an individual basis or, if the *navigate by genome* option is being used, for all proteins in a specific genome. Individual searches are initiated with the ‘Run’ button in the result visualization pane (Fig. 1E). The tool to use and the database to search against can be selected with the buttons at the top of the navigation pane. If the *navigate by genome* option is used, there will be an additional pane below the navigation pane which will allow users to select tool/database combinations for searching every protein in the genome (Fig. 1C).

After search results are returned to the server, they can be viewed in the result visualization and result table. The result visualization depicts how each protein returned from the database aligns to the query, showing where the alignment starts and stops on the query and the statistical significance of each alignment (Fig. 1E). Further information about each result is displayed in the result table (Fig. 1F).

The results visualization and table provide the users with information so they can fill out the annotation form (Fig. 1D). The annotation form provides fields for the *Annotation* label, the *Flag, Public notes*, *Private notes*, and the *Assigned to* field. The *Flag* field provides a way for users to sort annotations into groups. Annotations flagged with a color will appear that color in the genome visualization. Color flags have no predefined meaning; therefore what each color represents can be determined by the user/lab. There are also several predefined flags such as the ‘UNANNOTATED’ flag, which is assigned to newly created CDS annotations and the ‘REVIEW NAME’ flag, which is available to signal that the annotation needs to be revisited. The notes fields provide a way for users to describe their findings in greater detail. *Public notes* will be shown in the GenBank file created from the ‘Export genome data’ button, while *private notes* will not. The *Assigned to* field allows for adding the annotation to a user queue so it will show up in that user’s assignment navigator.

### Tools and databases

There are currently three homology search tools implemented in MAS: BLASTp, rpsblast (Reverse PSI-BLAST), and HHsearch. BLASTp allows users to search for primary protein structure homology [21]. MAS includes three blastp databases by default: SWISS-PROT, NCBI’s non-redundant (nr) protein database, and an internally generated database. SWISS-PROT is a manually curated database which strives to achieve high levels of annotation and minimal redundancy [22]. SWISS-PROT is a relatively small database but it contains highly accurate information. The nr protein database is massive and contains various levels of curated data from multiple sources such as RefSeq, UniProtKB, Protein Data Bank (PDB), Protein Research Foundation (PRF), and CDS translations from all proteins in GenBank [23]. The internal blast database is a database automatically generated by MAS. It includes the protein translations for all CDS annotations within the local MAS installation. It automatically updates itself when an annotation is edited or when a new genome is uploaded and supports consistency and self-review in the manual annotation process.

MAS uses rpsblast to search for homologous domains [21]. Rpsblast can be used to search against the Conserved Domain Database (CDD) [24]. The CDD contains protein domain models from SMART [25], Pfam [26], TIGRFAMS [27], COG [28], ProtClustDB [29], NCBIfam [30], and CDD’s own curation efforts. It currently contains over fifty-five thousand protein and protein-domain models.

HHsearch allows users to search for distantly homologous proteins by taking a multiple sequence alignment (MSA) as input and searching a database of hidden Markov models (HMMs) [31]. MAS is able to use HHsearch to search for homology against a specially compiled version of PDB. PDB contains atomic level structures of proteins, DNA, and RNA. These structures have been experimentally derived from X-ray crystallography, nuclear magnetic resonance spectroscopy, or three-dimensional electron microscopy. It currently holds over 155,000 models [32]. MAS uses a version of PDB, released by the maintainers of HH-Suite, that is clustered to 70% maximum pairwise sequence identity. MAS uses HHblits to create the MSA used as input into HHsearch. HHblits builds an MSA from the query sequence by iteratively searching the Uniclust30 database. Uniclust30 contains UniProtKB sequences clustered at 30% pairwise sequence identity [33].

### Additional capabilities

**Fig. 2:**
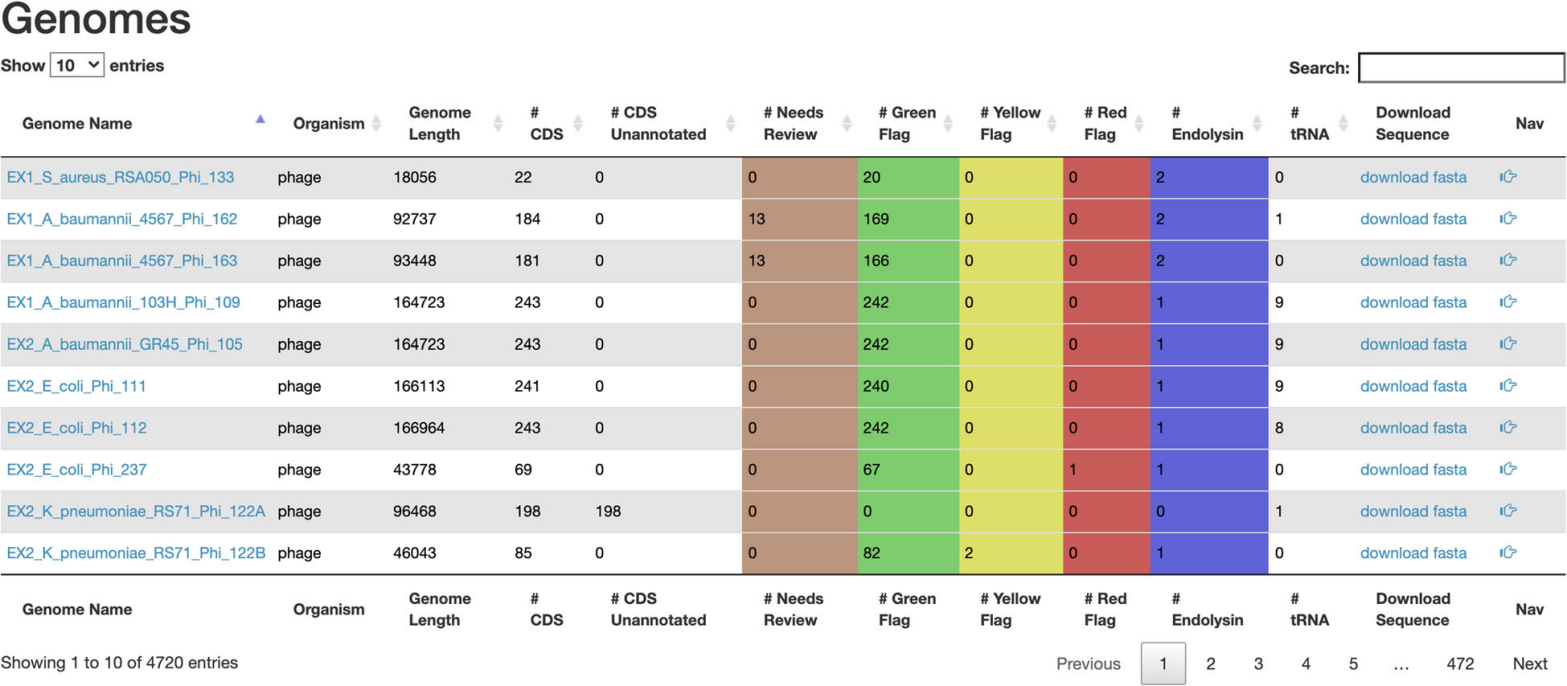
The genome list. This list displays information about all genomes that have been uploaded to MAS. From this page, users can navigate to the details page of any genome, navigate to the result viewer for any genome, filter the data displayed with the search bar, download genome sequences as a FASTA file, and sort by column. This also provides a way of quickly identifying which annotation flags each genome contains

Users can view a list of previously uploaded genomes or annotations through the genome list (Fig. 2) and annotation list tabs. Both these lists can be filtered with a search query and ordering can be changed based on selected columns. Relevant information and links to relevant pages for each genome/annotation are presented in tabular format. The annotation table provides a drop-down pane to present the annotation’s sequence in FASTA format.

Each genome in MAS has its own details page which displays summary information and lists of all features and annotations found within that genome. From here, users may download a FASTA file for the genome or use the ‘export genome data’ option to download a tarball containing a FASTA file for the genome sequence, a multi-FASTA file containing translations of the genome’s CDSs, a general feature format (GFF) file describing the genome’s features, and an excel document listing the genome’s features and annotations.

MAS provides a way for users to easily upload annotations produced outside of MAS through the ‘upload annotations from excel’ tab. The label, notes fields, and flag described in each row of the uploaded excel file will be matched to an existing annotation in the database by protein sequence.

### Usage example

In this section, we illustrate how a general user could leverage the capabilities of MAS for their project by walking through an example use case. MAS was originally created to support bacteriophage genome annotation but has been extended for use in other organisms. In this example, we show how MAS can be used to characterize the genomes of bacteriophages which are candidates for use in phage therapy, i.e. phages which may be used to treat antibiotic resistant bacterial infections.

#### Motivation

It is important to have a large and diverse set of phages available in order to effectively treat a wide range of bacterial infections because phages are specific to their bacterial host. Subsequently, this raises a safety concern and it is likely not feasible to perform in vitro safety tests on all phages. Genomic sequencing and characterization offers a more achievable alternative to in vitro tests. Confidence in the safety of a phage can be strengthened by screening its genome for genes with potentially harmful functions. In this case, genes with harmful functions would include genes that increase bacterial virulence, antibiotic resistance (AR) genes, and genes which indicate a lysogenic lifestyle. The use of MAS would be beneficial in this situation for a variety of reasons:

- MAS excels at the annotation of novel genomes. Phage genomes are extremely diverse and many newly sequenced phages are highly novel. This means that the functions of genes within the genome will have to be predicted based on distant homology to previously characterized genes. Many sequence homology tools and automated annotation tools are not sensitive enough to detect distant homology. The HH-suite of tools, which contains HHsearch, was built to detect distant homology. To our knowledge, MAS is the only annotation editing tool which contains the tool HHsearch.
- MAS can annotate genomes with a high degree of accuracy. In this situation, the accuracy of annotations is critical because failure to detect a problematic gene could adversely affect a patient’s health outcome or response to treatment. Annotations based on a single database are highly sensitive to, or biased by, poorly annotated sequences contained within that database. MAS allows users to compare homology search results across different tools and databases, giving users a better chance at detecting erroneous results.
- MAS can assist with the categorization of annotations. In this example, the user will make a determination on whether a gene is problematic or not during the annotation process. MAS can help users make this determination by providing evidence and by allowing them to assign annotations to categories through annotation flags.

The same reasons for using MAS apply to other situations where annotation accuracy is critical, the genome is novel, or it is necessary to categorize a diverse set of genes.

#### Preparing data for MAS

To start using MAS, the user must first generate and prepare a genome sequence. This genome sequence can be created from raw sequencing data through de novo assembly or reference-based approaches [34]. Ideally, the user will generate a complete genome sequence or chromosome. Doing so may require the use of genome finishing techniques [35]. With respect to phage genomes, this would entail removal of assembly artifacts and genomic termini resolution [20].

#### Uploading the genome

Once the user has a genome sequence in FASTA format, they can start using MAS to annotate it. Before annotation can occur, the genome must be uploaded to MAS. The user has a number of options available at this stage. In most situations, the user will opt to allow MAS to automatically determine the location of genes in the uploaded sequence. MAS accomplishes this using GLIMMER and tRNAscan-SE [1, 36]. The user also has the option to perform gene-calling outside of MAS using a different automated gene-caller or a tool such as DNA master to manually determine gene locations [37]. In the latter situation, the user will upload the FASTA file using the “Upload Custom Genome” option whereas in the former situation, the user will upload the FASTA file using the “Upload Phage Genome” option. The physical orientation of a phage’s chromosome is dependent on its packaging strategy [38], however, for many species of phages, this orientation is random between individuals. If this is the case, the user will allow MAS to automatically reorient the uploaded genome sequence to start from the terminase gene. This will facilitate comparative analysis between similar genomes. In other cases, the phage genome is flanked by direct repeat sequences. The user will provide the length of these sequences during genome sequence upload in order to allow MAS to annotate them automatically. The user also must select a name for the genome during the upload process. When naming a phage genome in MAS, the selected name must be unique and it is best practice to include information about the host bacterium. The user may also wish to include additional information in the genome name such as a project identifier, the sample source location, or the name of the laboratory that collected/sequenced the sample. Including this information in the genome name allows the user to query genomes based on it.

#### Annotation

To begin the annotation process, the user selects the homology search tools and databases they would like to use and initiates the searches for each CDS. If not limited by computational resources, the user may initiate searches for all available tools and databases. The searches will execute in the background and as soon as each individual search finishes, results will populate for the user to review. As results are becoming available, the user will navigate through the CDS in the uploaded genome, viewing the returned results for one at a time. As described in the previous Tools and Databases section, the user will have access to results from three homology search tools: BLASTp, HHSearch, and RPS-BLAST. HHsearch will be used to search the PDB, RPS-BLAST will be used to search against the CDD, and BLASTp will be performed against three separate databases: SWISS-PROT, NCBI’s nt, and an internally generated BLAST database. The results for all of these tools will be accessible through a single webpage. The user will review these results to determine a name for the annotation, what notes to include, and what flag to assign to it. There are multiple things to consider in order to assign the best possible annotation. The user must consider which tools and databases returned results, the significance of the results, length of the result sequence, consistency among results, length of the alignment, and position of the alignment with respect to both the query sequence and the result sequence. In general, a good strategy is to quickly run-through the results from each database to see which tools returned statistically significant results. Once the user knows which results to consider, they can interrogate the results further and compare results across databases. The process of determining the best label for an annotation through the interrogation of results in MAS is summarized in Table 1.

**Table 1:** A general guide for using results in MAS to determine an annotation label. This guide is meant to serve as an example for how a user could interrogate results and how a user could proceed in a variety of different situations. Each row represents a particular situation, which leads to a procedure. Depending on the results, one or more procedures may be applicable. Grey cells in a row mean the question is non-applicable or not considered

Has BLASTp vs SP results?	Has BLASTp vs. internal DB results?	Has HHSearch Results?	Has RPS-BLAST Results?	Has BLASTp vs. nr results?	Are the results significant?	Are Results Consistent Across Databases?	Are Results Consistent for a Specific Database?	Is the length of the result protein / domain similar to the query?	How much of the result sequence does the alignment cover?	How much of the query sequence does the alignment cover?	Procedure
No	No	No	No	No							Label as ‘hypothetical protein’.
					No						
No	No	No	No	Yes	Yes		No				Results can typically be disregarded and the protein can be labelled as ‘hypothetical protein’. If there is a particular interest in the query protein, examine results in greater detail to determine if they are reliable.
No	No	No	No	Yes	Yes			No			
No	No	No	No	Yes	Yes		Yes	Yes			Look further into nr results to determine if they are reliable. Look to see if the results are supported by literature or if there was another tool/database used.
No	No	No	Yes		Yes		Yes	Yes	All/Most	All/Most	If the result decribes a singular function, such as the “Phage terminase large subunit (GpA)” domain included in CDD, label the protein according to the function. If the result does not describe a meaningful function, such as one of CDD’s domains of unknown function (DUF), mark the protein as ‘XXX domain-containing protein’, where XXX is replaced by the name of the domain.
No	No	No	Yes		Yes		Yes	No	All/Most	Some	Mark the protein as ‘XXX domain-containing protein’, where XXX is replaced by the name of the domain.
No	No	No	Yes		Yes		No	No	All/Most	Some	If the inconsistent domain hits align to the same location in the query, mark the protein as ‘XXX domain-containing protein’, where XXX is replaced by the name of the most significant domain. If inconsistent domain hits align to separate locations in the query, the query likely contains multiple domains. If a name can describe the function of all or a majority of the domains, that name should be used. Otherwise, consider the functional importance of the domains and statistical significance of the results to select a single domain to name the annotation with.
Yes	No	No	No		Yes		Yes	Yes	All/Most	All/Most	SwissProt results are generally trustworthy and can be used directly to annotate your query.
Yes	No	No	No		Yes		No				Confirm accuracy of the SwissProt annotations by using the link in the results table to view the entry within SwissProt.
Yes					Yes		Yes	No	All/Most	Some	If the result protein is known to have homologs which are functionally similar but differ in length, the result may still be trustworthy. If the length of the result and query differ by more than an expected amount, examine the position of the alignment in the query. If the result aligns to the C-terminus of the query, the start codon may be incorrect. If you are annotating a viral protein, consider if this is a polyprotein.
	Yes										
		Yes									
Yes					Yes		Yes	No	Some	All/Most	If the result protein is known to have homologs which are functionally similar but differ in length, the result may still be trustworthy. If the length of the result and query differ by more than an expected amount, examine the position of the alignment in the result protein. If the query aligns to the C terminus of the result, the start codon in the query may be incorrect and the true start codon may be upstream. If the query aligns to the N-terminus of the result, the query may be truncated. This can be caused by a mutation or a transposable element. If the start codon is correct, examine the result protein to see if the location of the alignment represents a functional domain.
	Yes										
		Yes									
Yes					Yes				Some	Some	Examine the location of the alignment within the result protein and query. The result protein and query may share a functional domain.
	Yes										
		Yes									
Yes	No	No			Moderate Significance		Yes	Yes	All/Most	All/Most	When only a moderate level of significance is exhibited for the results and there is not confirmation with multiple databases and tools, consider prepending the annoation label with “putative”.
No	Yes	No									
No	No	Yes									
No	Yes	No	No		Yes		Yes	Yes	All/Most	All/Most	Review the result protein in MAS to confirm the accuracy of the annotation.
No	No	Yes	No		Yes			Yes	All/Most	All/Most	Since HHSearch is more sensitve than the other tools, extra care needs to be taken to not overannotate the query protein with a highly specific function based on its results. For example, a result protein may be labelled as a specific type of nuclease, such as a cell death-related nuclease. If you only have HHSearch results to base your annotation on, it is safer to label the query annotation in a more general manner, i.e. ‘nuclease’. When it is not clear how to generalize the annotation, consider prepending the annoation label with “putative”.
Yes	Yes				Yes	Yes	Yes	Yes			Base the labelling of the annotation on the results from the internal database so naming conventions remain consistent.
	Yes	Yes									
	Yes		Yes								
Any combination of 2 or more					Yes	No					Investigate the cause for the inconsistency. Consider the evidence for competing results. Which result has a lower e-value? Which database is is more higly curated? View the result protein within its native database to see what evidence is given to support the given annotation.

In addition to the annotation’s label, the user will decide which flag to assign to the annotation. The Flag field provides the user with a way of categorizing annotations. Some of the provided flag options have defined meanings. For example, the ‘review name’ flag signifies that the annotation needs further review and the ‘tRNA’ flag is automatically assigned to tRNA genes discovered by tRNAscan-SE. The user can also assign the annotation to a color using the flag field. The meaning of each color is left to the user’s discretion. In our example, the user could assign color flags as a way of categorizing proteins according to risk. Proteins that would not adversely affect the efficacy or safety of phage therapy would be assigned the ‘green’ flag, while proteins which would have an adverse effect, such as an integrase gene or a toxin, would be assigned the ‘red’ flag. The color of each flag is reflected in the genome visualization, users can search for annotations by their flag, and genomes can be sorted according to the number of proteins assigned to each flag. In addition, each protein’s flag will be saved in the internal BLAST database, aiding future annotation. Any important information that is not described by the annotation label or flag can be added to the notes fields. When the user finishes annotating a phage genome, the can export the phage’s data in multiple formats using the “download deliverables” option on the phage details page.

### Future improvements

MAS will serve as a platform for further development, with additional tools, databases, and features added to support the varying needs of its users. Planned future improvements include additional gene callers, improved genome visualizations, transmembrane domain prediction, rRNA prediction, additional feature types, custom flags, and support for annotating alternative splicing and polyproteins.

## Implementation

### General overview

**Fig. 3:**
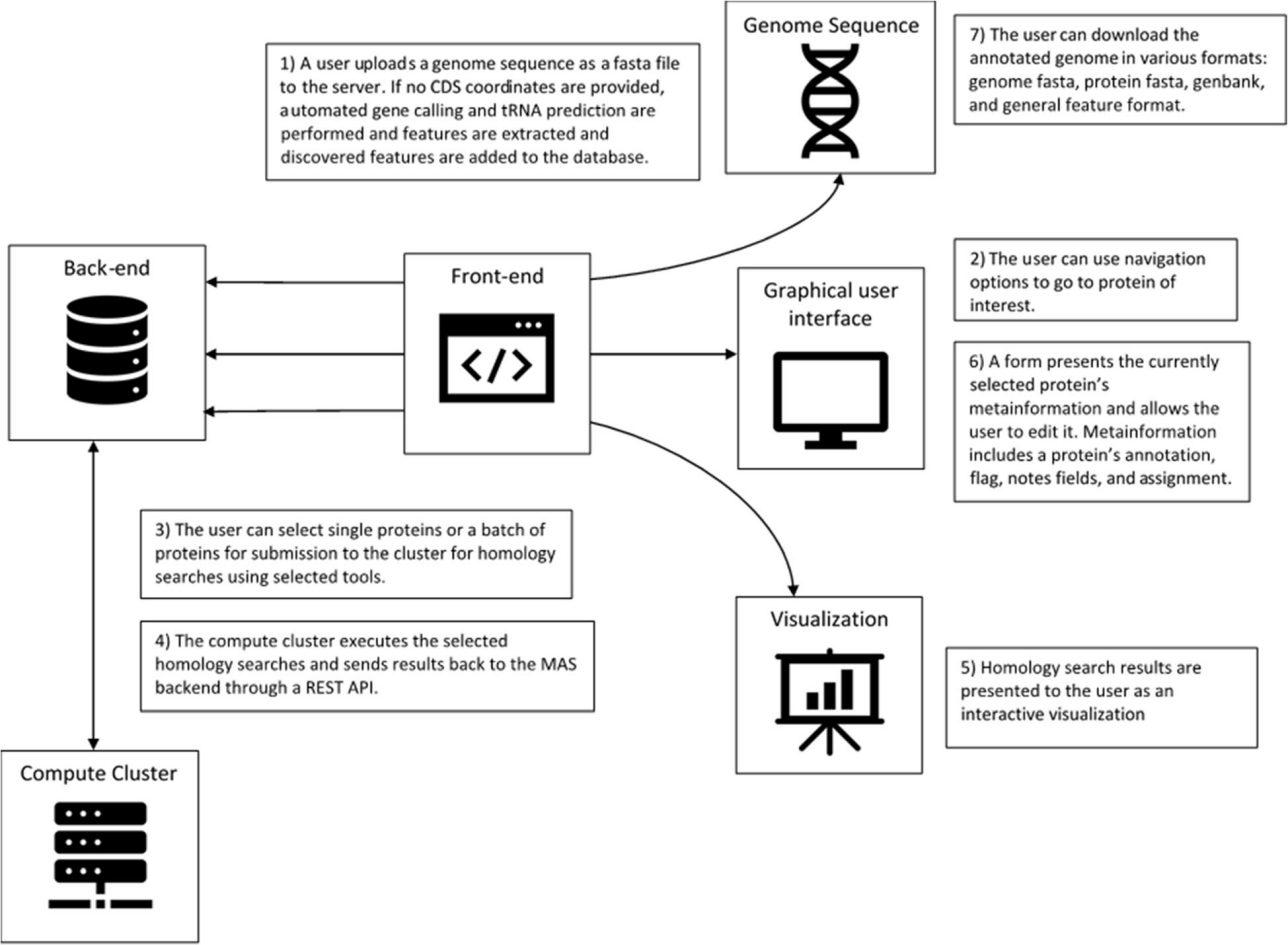
A simplified overview of MAS. Users start by uploading a genome sequence. Then, they initiate run homology searches. When search results are ready, users can start the annotation process by viewing results and submitting annotation information through the provided form. When users finish annotating a genome, they can export it in a variety of formats

MAS was designed to run as a local web-server allowing multiple users to simultaneously interact through a web-browser and to collaborate by accessing and updating shared data. The annotation process starts when a user uploads an unannotated genome in a FASTA formatted file (Fig. 3). The user can also provide the coordinates of coding sequences (CDS) within the genome, or MAS can automatically predict their locations. The genome and features (such as coding sequences) are stored in a database. The user can then initiate a variety of homology searches using the graphical user interface (GUI). Homology search results are also stored by MAS and are presented to the user in a graphical representation and in a result table. The user can then examine these results to determine how to annotate the associated protein. Annotation information is provided to MAS through a form and is saved to the database upon submission. Users can navigate information stored in the database through a variety of views and information can be exported from the database in a variety of formats.

### Architecture

MAS consists of multiple interacting components which work together to provide a functional webserver (Supplementary Figure 1). Most of these components are containerized using Docker. MAS uses Docker to make installation and updates easier for users and to provide enhanced portability. There are three Docker containers that are built and run through the docker-compose command: a container housing the Apache server, a container housing the SQL server, and a container for the message broker. The fourth and final component of the MAS system is the worker, which is installed directly on the host machine and is responsible for handling computationally intensive tasks.

The MAS server uses the Django (v3.1) web framework and is written in Python (v3.8), JavaScript, and HTML. The Django/Apache server container is built from a Dockerfile using the official Docker Apache httpd image as a base image. Interactive visualizations are created within JavaScript using the D3.js library. The Biopython python library [39] is used to parse FASTA files and results from homology searches. The homology search pipeline uses the Luigi framework in order to help manage long running batch processes. With a default configuration, the MAS website uses port 8080 and the Luigi scheduler uses port 8082. Homology search results are stored as text files by the server to a Docker volume.

Uploaded genomes, genomic features, annotations, homology search result metadata, and user information are stored within a MariaDB SQL server (Supplementary Figure 2). The SQL server resides in a Docker container built from the official MariaDB Docker image. Data stored to the database is written to a Docker volume, which allows the data to persist when the SQL server container is destroyed or rebuilt.

MAS allows users to trigger long running homology search pipelines. These pipelines are activated through the Celery distributed task queue allowing them to be executed asynchronously by the worker, outside of the HTTP request/response cycle. The MAS server communicates tasks which need to be executed to the worker through a RabbitMQ message broker. The worker can then directly execute the task, or if the task involves a pipeline of multiple long-running batch process, it can initiate a Luigi pipeline. Luigi pipelines are initiated by the worker when users submit homology searches to the server. These pipelines can execute searches directly on the host machine or in a high-performance computing (HPC) environment through SGE or SLURM batch-queuing systems depending on how MAS was configured during the installation process. Homology search pipelines communicate results back to the server through a REST API. The worker is installed directly on the host machine, rather than in a Docker container, in order to enable cluster compatibility.

## Conclusion

MAS provides researchers a way to streamline the process of manual functional annotation. Running homology searches in MAS requires less overhead than running the searches through online services and less bioinformatics expertise than running searches through the command-line. MAS facilitates reproducibility by saving search results, annotations, and annotation history in a structured database. Team annotation efforts benefit from the user management system MAS provides and from its ability to allow users to view and edit shared data concurrently. We will continue to work towards integration of additional tools and databases into MAS in order to provide annotators with additional information and visualizations to improve the quality of their annotations even further.

## Availability and requirements

Project name: Manual Annotation Studio

Project home page: https://github.com/BDRD-Genomics/MAS

Operating System(s): Server: Linux and OSX, User: Platform independent

Programming languages: Python, JavaScript, HTML, CSS

Other requirements: Docker, Conda package manager, modern web browser

License: GNU General Public License v3.0

Any restrictions to use by non-academics: None

## Supplementary Information

**Additional file 1: Supplementary Figure 1.** The architecture of MAS. Installation of MAS will automatically build three Docker containers: 1) a Docker container containing MAS’s code, the Apache server, and the Luigi daemon, 2) a Docker container containing the database server, and 3) a Docker container containing the message broker used to send tasks to the MAS Worker. These three Docker containers exist under the same Docker engine and communicate with each other through a bridge network. Computationally intensive jobs are sent to the MAS worker using the Celery distributed task queue. The worker can execute the workload on the host machine or on a compute cluster through either the SLURM or SGE batch-queuing systems.

**Additional file 2: Supplementary Figure 2.** MAS Database Schema.

## Data Availability

MAS source code is available on GitHub, https://github.com/BDRD-Genomics/MAS.

## Abbreviations

API: Application programming interface
CDD: Conserved Domain Database
CDS: Coding sequences
GFF: General Feature Format
GUI: Graphical User Interface
HMM: Hidden Markov Model
HPC: High-Performance Computing
LAN: Local Area Network
MAS: Manual Annotation Studio
NR: Non-Redundant
PDB: Protein Data Bank
PGAP: Prokaryotic Genome Annotation Pipeline
PRF: Protein Research Foundation
RAST: Rapid Annotation using Subsystems Technology
SLURM: Simple Linux Utility for Resource Management
