# Supplementary figures and images for: Manual Annotation Studio (MAS): a collaborative platform for manual functional annotation of viral and microbial genomes
Source: BMC Genomics. 2021 Oct 9;22:733. doi: 10.1186/s12864-021-08029-8 (PMC8501643; doi:10.1186/s12864-021-08029-8)

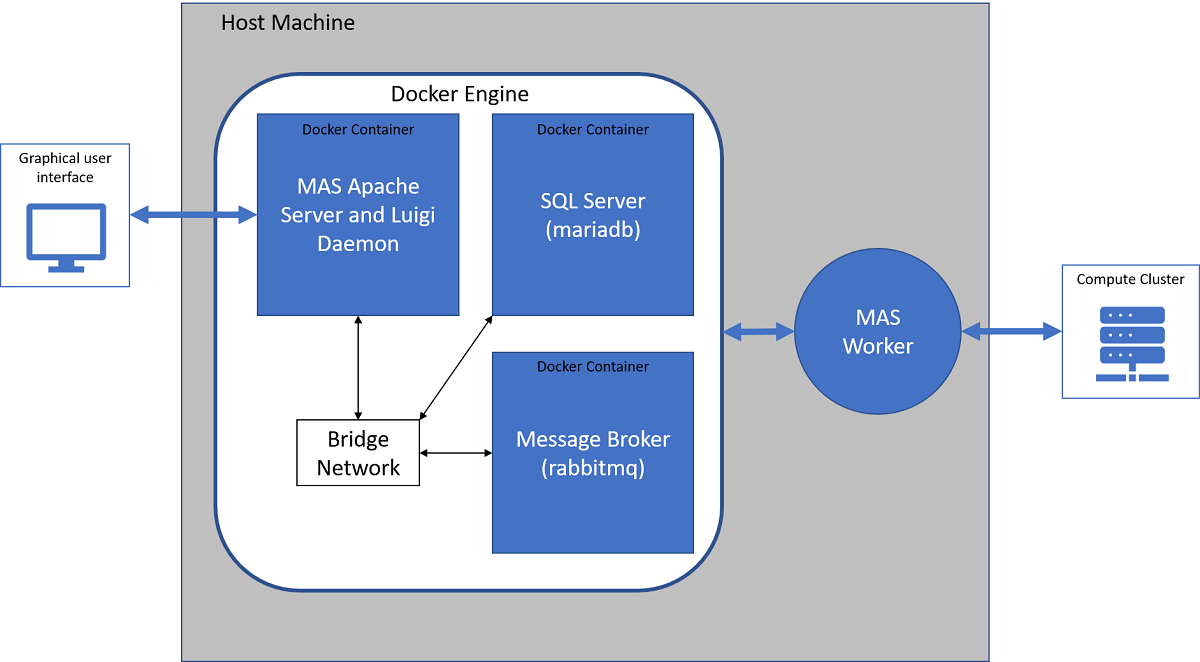

Supplement: Supplementary file 1 — Additional file 1: Supplementary Figure 1. The architecture of MAS. Installation of MAS will automatically build three Docker containers: 1) a Docker container containing MAS’s code, the Apache server, and the Luigi daemon, 2) a Docker container containing the database server, and 3) a Docker container containing the message broker used to send tasks to the MAS Worker. These three Docker containers exist under the same Docker engine and communicate with each other through a bridge network. Computationally intensive jobs are sent to the MAS worker using the Celery distributed task queue. The worker can execute the workload on the host machine or on a compute cluster through either the SLURM or SGE batch-queuing systems. [file 12864_2021_8029_MOESM1_ESM.png]

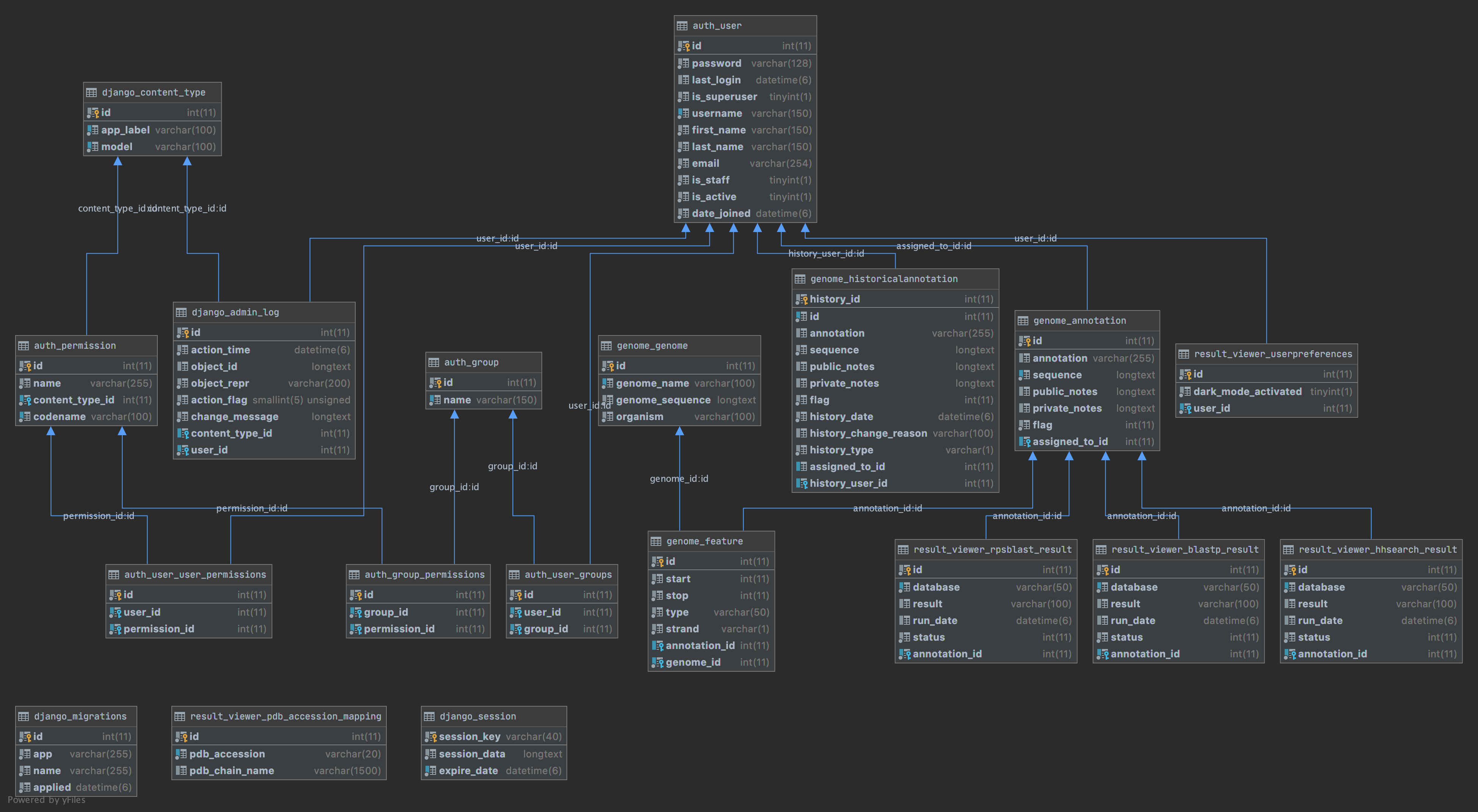

Supplement: Supplementary file 2 — Additional file 2: Supplementary Figure 2. MAS Database Schema. [file 12864_2021_8029_MOESM2_ESM.png]
